# Effects of Dietary Supplementation with Combination of Tributyrin and Essential Oil on Gut Health and Microbiota of Weaned Piglets

**DOI:** 10.3390/ani10020180

**Published:** 2020-01-21

**Authors:** Wen-Xin Zhang, Yu Zhang, Xiao-Wei Zhang, Zhao-Xi Deng, Jian-Xin Liu, Mao-Long He, Hai-Feng Wang

**Affiliations:** 1 College of Animal Science, Zhejiang University, Hangzhou 310058, China; zhangwenxin1002@163.com (W.-X.Z.); soaryunov@163.com (Y.Z.); zhaoxideng@zju.edu.cn (Z.-X.D.); liujx@zju.edu.cn (J.-X.L.); 2 Animal Husbandry Technology Promotion Station of Zhejiang Province, Hangzhou 310021, China; xmzxxmzx@tom.com; 3 Innovation Division, Lucta (Guangzhou) Flavours Co. Ltd., Guangzhou 510530, China; maolong.he@lucta.com

**Keywords:** tributyrin, methyl salicylate, oregano, gut, microbiota, piglets

## Abstract

**Simple Summary:**

The dietary inclusion of the combination of tributyrin with either oregano or methyl salicylate as a substitution to antibiotics improved intestinal morphological structure of weaned piglets and resulted in major changes in the profiles of intestine microbiota and metabolites, which exerted beneficial effects on intestinal health of piglets. Our study indicated that the combination of tributyrate with oregano or methyl salicylate could be used as an alternative feed additive to the antibiotics.

**Abstract:**

The aim of this study was to determine the effects of dietary inclusion of the combination of tributyrin with oregano or methyl salicylate as a substitute to antibiotics on gut health and microbiota of piglets. A total of 48 weaned crossbred piglets (Duroc × Large White × Landrace, 8.79 ± 0.97 kg, 21 ± 1 d) were randomly allocated to four experimental groups and fed for 4 weeks: the basal diet (Con); the control plus antibiotics (AB); the control plus oregano and tributyrin (OT); and the control plus methyl salicylate and tributyrin (MT). Although a numerical improvement on feed intake, weight gain and feed conversion ratio was observed in the OT and MT as well as the AB group, the difference was not significant (*p >* 0.05). The OT and MT groups were larger in villus height in the duodenum compared to the Con (*p <* 0.05), and were larger in relative abundance of *Firmicutes*/*Bacaeroides* in the intestine compared to Con and AB groups (*p <* 0.01). The amount of major different metabolites was 6, 8 and 8 for the AB, OT and MT groups when compared to the Con, respectively. In conclusion, as a substitute for antibiotics the inclusion of the combination of tributyrin with either oregano or methyl salicylate to the diet of weaned piglets improved the intestinal morphological structure and altered intestinal microbiota and metabolites, which were beneficial to the animal health.

## 1. Introduction

Antibiotics such as tetracycline have been used as feed additives to prevent diarrhea, promote animal growth and improve feed utilization in piglets. However, overusing antibiotics in animal feed results in residues in products and causes antibiotic resistance to pathogenic bacteria [1]. Therefore, it is necessary to find proper alternatives as feed additives to the antibiotics currently used for improving intestinal health and relieving the weaning stress in piglets [2,3]. Plant extracts and essential oils might be used as alternatives to antibiotic growth promoters through improving gut health [4,5]. Tributyrin (TB) contains three butyric acid moieties esterified to a glycerol backbone [6]. On a molar basis, when hydrolyzed by cellular lipases or esterase, TB could be3-fold more potent than butyrate, which could result in a higher butyrate level and keep longer in serum as compared with butyrate salts [7,8,9]. TB is well tolerated orally and has been approved as a dietary additive in the United States and many other countries [10]. Previous studies found that as a short-chain fatty acid (SCFA), butyric acid could be beneficial to intestine health by inhibiting the growth of bacteria, increasing mucosal cell proliferation, and improving intestinal cell development, as well as colonic defense work [11]. Oregano is a perennial herbaceous plant of the genus *Oryx* [12]. As a plant extract, oregano oil is mainly composed of phenolic compounds including carvacrol, thymol, cymene and terpinene [13]. Previous studies found that oregano oil could prevent diarrhea in weaned pigs caused by *Escherichia coli* and improve overall body weight gain and health [14]. Methyl salicylate is also an essential oil extracted from the leaves of holly and was found to have bactericidal and bacteriostatic effects in previous studies [15]. However, little information was available on the effect of methyl salicylate on the intestine health and growth performance of weaning piglets.

Based on our previous in vitro studies on the effect of various combinations of tributyrin and essential oils against *Escherichia coli*, *Salmonella* and *Staphylococcus aureus* [16], two combinations were selected for the present animal feeding trial. This study was designed to test the hypothesis that dietary supplementation of tributyrin and oregano or tributyrin and methyl salicylate could exert beneficial effects on the intestine health of weaned piglets through both inhibiting *Escherichia coli* and promoting mucosal cell proliferation. The major investigated effects included those on the ratio of villus height/crypt depth in the intestine, general health-related blood parameters, and intestinal microbiota and metabolites.

## 2. Materials and Methods

### 2.1. Animals and Experimental Design

All animal procedures were approved by the Animal Care and Use Committee of Zhejiang University, China (ethics code permit no. ZJU20170529). A total of 48 piglets (Duroc × Large White × Landrace,21 ± 1 d) with an averaged body weight of 8.79 ± 0.97 kg were selected and randomly allocated to one of 4 experimental diets with three pens, with 4 piglets for each pen (12 piglets/treatment in three pens): (1) control diet (Con), which was an antibiotic-free basal diet composed based on the nutrition standard for weaned piglets of the National Research Council (NRC) [17]; (2) the basal diets supplemented with the antibiotic compounds (AB), with 120 mg/kg oxytetracycline-calcium and 16 mg/kg enduracidin; (3) the basal diet supplemented with oregano plus tributyrin (OT) in a dose of 1.4 g and 0.6 g per kilogram diet, respectively; (4) the basal diet supplemented with methyl salicylate plus tributyrin (MT) in a dose of 1.4 g and 0.6 g per kilogram diet, respectively. The test samples of oregano, methyl salicylate and tributyrin were products of Lucta (Guangzhou) Flavours Co., Ltd. The composition of diets and the nutrient concentration are shown in Table 1. The feeding trial lasted for 4 weeks. The piglets had free access to feed and drinking water. The piglets were weighed individually, at the start and at the end of the experiment. Daily feed intake of the piglets in the pen was measured.

### 2.2. Blood and Intestine Sample Collection and pH Measurement

At the end of the trial, 6 piglets form each treatments (3 males and 3 females) were euthanized to collect blood samples, tissue samples from the duodenum and ileum, and the contents of the ileum and colon. The segments of the duodenum and ileum were taken as specimens and fixed with phosphate-buffered paraformaldehyde (4%, pH 7.6) for further histological measurements. The pH value of the contents in the stomach and intestine were determined.

### 2.3. General Health and Anti-Oxidative Capacity Related Blood Parameters Assays

General health and anti-oxidative capacity related blood parameters included serum total protein, albumin, glucose, blood urea nitrogen (BUN), malondialdehyde (MDA) and activities of alkaline phosphatase (ALP), alanine aminotransferase (ALT), aspartate aminotransferase (AST), glutathione peroxidase (GSH-Px), superoxide dismutase (SOD), and oxidation resistance (T-AOC). They were determined by kinetics-based assays with commercially available kits (Nanjing Jiancheng Bioengineering Institution, Nanjing, China) using an automatic biochemistry analyzer (SELECTA XL; Vital Scientific, Newton, MA, USA) according to the protocol provided by the manufacturer.

### 2.4. Intestinal Morphology Analysis

Hematoxylin-eosin (H&E) staining was performed as previously described [18]. Intestinal samples from the piglets were soaked in 10% neutral paraformaldehyde and covered with wax. The waxed tissue blocks were sliced manually into 3-μm-thick sections and were treated for deparaffinization and dehydration. The sections were then treated with a series of graded alcohols (100%, 95% and 75%) for 15 min each and subsequently stained with H&E. Photomicrographs were obtained using an optical microscopy system (Olympus Corporation, Tokyo, Japan). For each section, 10 fields were examined using a light microscope with a computer-assisted morphometric system. Intestine villus height and the corresponding crypt depth were measured.

### 2.5. Short-Chain Fatty Acid Analysis

Short-chain fatty acids in the samples were analyzed based on a previous method with minor modifications [19]. Briefly, the colon content sample was mixed with deionized water in a ratio of 1 g/mL and vortexed for 30 s before being centrifuged at 15,000× *g* at 4 °C for 15 min. The supernatant was transferred to a new tube, then 25% metaphosphoric acid was added in at the ratio of 9:1 (v/v). The VFAs concentration in the supernatant was determined using a gas chromatograph (model number: GC-2010; Shimadzu Corp, Kyoto, Japan) equipped with a column (HP-INNOWAX(19091N-133), 30 m × 0.25 mm × 0.25 µm).

### 2.6. Counting of Escherichia Coli and Lactic Acid Bacteria inFresh Fecal Samples

The numbers of *Escherichia coli* and lactic acid bacteria were determined based on previous methods [20,21] with minor modifications. In brief, a fresh fecal sample (1 g) was 10-fold gradient diluted with sterile physiological saline. MRS agars and EMB were used for determination of lactic acid bacteria and *Escherichia coli*. The plates were incubated at 37 °C for 24 h. The microbial enumerations of digesta were counted and expressed as colony-forming units per gram sample.

### 2.7. Bacterial 16S rRNA Sequencing and Bioinformation Analysis

High-resolution 16S rDNA genes of the bacterial community in the colonic content were analyzed based on a previous method with modifications [22]. The hypervariable V3–V4 region was amplified to obtain the original sample library. The library was sequenced using the two-terminal sequencing strategy of 250 bp on the Miseq platform, followed by bioinformatics analysis. Chimeric sequence detection and de novo operational taxonomic units (OTU) picked up with 0.97 identities were implemented using USEARCH and UCLUST algorithms, respectively. Taxonomy assignment of representative sequences from each OTU was performed using the Ribosomal Database Project classifier against its reference database with confidence cut off of 0.8.

### 2.8. GC-TOF-MS and Metabolomics Data Analysis

Fecal samples were lyophilized, derivatized and analyzed by gas chromatography time-of-flight mass spectrometry (GC-TOF-MS) (Agilent 7890 gas chromatograph and LECO Pegasus III time-of-flight mass spectrometer) as previously described [23,24]. MS-DIAL software [25] and FiehnBin base database were used for raw peaks exacting, the data baselines filtering, and calibration of the baseline [26]. Peaks detected in ≤50% of QC samples or <50% samples of every group were removed, except QC group or RSD>30% in QC samples [27]. SIMCA-P v13.0 (Umetrics, Umea, Sweden) was used for partial least squares-discriminant analysis (PLS-DA) and orthogonal projections to latent structures-discriminant analysis (OPLS-DA). The first principal component of variable in importance projection (VIP) was obtained to refine the analysis. VIP > 1.5 was first selected as “changed metabolites”. Obtained metabolites were validated by searching in the Kyoto Encyclopedia of Genes and Genomes (KEGG) [28].

### 2.9. Statistical Analysis

All data were subjected to ANOVA analysis on the GLM procedures of SAS 9.1.3 (SAS Institute, Cary, NC, USA). Means were further compared by using Duncan’s multiple comparison when the variance derived from ANOVA was significant (*p* ≤ 0.05). An individual piglet was used as the statistics unit for the major data including blood parameters, intestinal pH, and morphology data. The growth performance data was analyzed to obtain preliminary results with pen as the unit.

## 3. Results

### 3.1. General Health-Related Blood Parameters and Performance of the Piglets

The experimental piglets were generally in a healthy condition and kept normal growth during the feeding trial. Although the present study was not mainly designed for testing effect on growth performance, the preliminary results found that there was a trend that supplementation of OT and MT as well as AB numerically increased the daily body weight gain, feed intake and the feed conversion ratio (Table 2). The differences among the groups were not statistically significant (*p >* 0.05). There was no significant difference in serum total protein, albumin, glucose, BUN, AST/GOT, ALT/GPT, ALP, SOD, MDA, GSH-PX, T-AOC among different treatments (*p >* 0.05, Table 3).

### 3.2. Gastrointestinal Content pH and Colonic Short-Chain Fatty Acid Profile

The piglets in the MT groups had a lower pH value in the ileum compared to those in the Con and the OT groups (*p <* 0.05). There were no significant differences in the pH value in other intestine sections among different treatments (*p >* 0.05) (Table 4). There was no significant difference in piglet colon content volatile fatty acid profile among the four treatments (*p >* 0.05) (Table 5).

### 3.3. Small Intestinal Morphology Analysis

**Figure 1:**
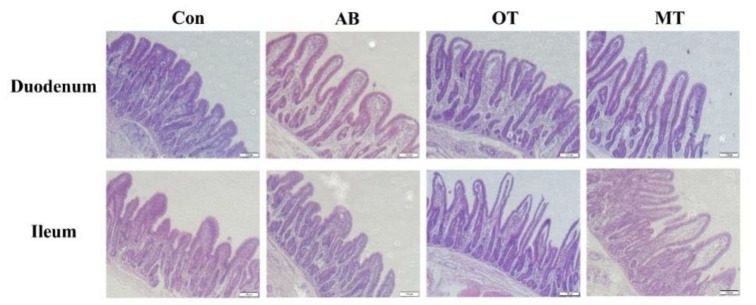
Histological and morphometrical analyses of small intestine by hematoxylin-eosin (H&E) staining. Con, the basal diet without antibiotics, tributyrin, oregano or methyl salicylate; AB diet, the basal diets supplemented with an antibiotic compound, which provides the diet with 120 mg oxytetracycline-calcium and 16 mg enduracidin per kilogram; OT and MT diets, the basal diet supplemented with tributyrin plus oregano (1.4 g and 0.6 g per kilogram diet) and tributyrin plus methyl salicylate (1.4 g and 0.6 g per kilogram diet), respectively. Villus height and crypt depth for the small intestine are in Table 4. The measurement of villus height (from the tip of the villous to villous-crypt junction) and crypt depth (from the villous-crypt junction to the lower limit of the crypt) were recorded as the mean of 10 fields for specimen. Scale bars: 100 μm.

The epithelial structures of the duodenum and ileum of piglets in each group appeared to be healthy (Figure 1). The intestinal mucosal tissues were intact without obvious pathological changes. In the duodenum, the dietary inclusion of OT and MT significantly increased villus height, and MT significantly increased the ratio of villus height to crypt depth compared with the control (*p <* 0.05) (Table 6). In the ileum, the AB group had significantly larger crypt depth compared to the Con, OT and MT groups, and lower ratio of villus height to crypt depth compared to the OT and MT groups (*p <* 0.05) (Table 6).

### 3.4. Numbers of Escherichia Coli and Lactic Acid Bacteria in Fresh Fecal Samples

**Figure 2:**
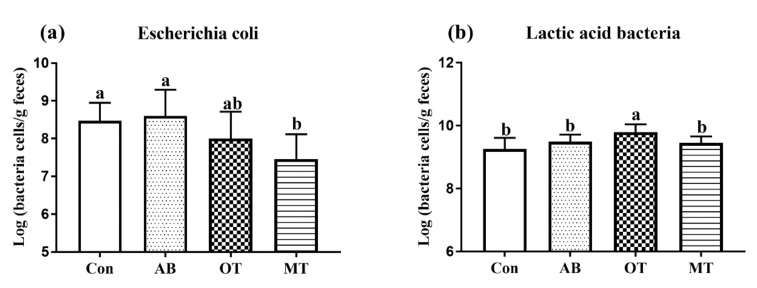
Counting of *Escherichia coli* and lactic acid bacteria in fresh feces of piglets. (**a**) *Escherichia coli*. (**b**) Lactic acid bacteria. Con, the basal diet without antibiotics, tributyrin, oregano or methyl salicylate; AB diet, the basal diets supplemented with an antibiotic compound, which provides the diet with 120 mg oxytetracycline-calcium and 16 mg enduracidin per kilogram; OT and MT diets, the basal diet supplemented with tributyrin plus oregano (1.4 g and 0.6 g per kilogram diet) and tributyrin plus methyl salicylate (1.4 g and 0.6 g per kilogram diet), respectively. One gram of the sample was weighed and diluted to a dilution of 10^−6^, 10^−7^, 10^−8^ by a 10-fold gradient dilution method. They were coated on EMB and MRS identification media for identification of *Escherichia coli* and lactic acid bacteria. Colony count = number of original bacterial colonies × dilution factor × 100 CFU/mL. ^a,b^ Means without a common superscript letter differ (*p <* 0.05) (*n* = 6).

The piglets in the MT group had significantly fewer fecal *Escherichia coli* than those in the Con group (*p <* 0.05) (Figure 2a). The piglets in the OT group had a significantly larger number of lactic acid bacteria in their feces compared to the piglets in the Con group (*p <* 0.05). The dietary inclusion of MT significantly reduced the number of *Escherichia coli* in the feces of weaned piglets compared to the AB treatment (*p* < 0.01) (Figure 2b).

### 3.5. Colonic Microbiota

**Figure 3:**
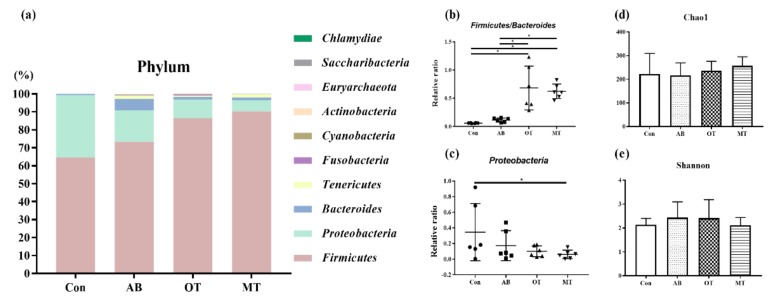
The high-throughput sequencing of 16S RNA in colonic feces of piglet. Con, the basal diet without antibiotics, tributyrin, oregano or methyl salicylate; AB diet, the basal diets supplemented with an antibiotic compound, which provides the diet with 120 mg oxytetracycline-calcium and 16 mg enduracidin per kilogram; OT and MT diets, the basal diet supplemented with tributyrin plus oregano (1.4 g and 0.6 g per kilogram diet) and tributyrin plus methyl salicylate (1.4 g and 0.6 g per kilogram diet), respectively. (**a**) Comparison of the phylum level between the 4 groups. (**b**) The relative ratio of the *Firmicutes* to *Bacteroidetes*. (**c**) The relative abundance of *Proteobacteria*. (**d**) Comparison of the abundance index (Chao1) between the 4 groups. (**e**) Comparison of the diversity index (Shannon) between the 4 groups. Error bars represent standard error of the mean. The *p*-values are based on Mann–Whitney tests. The statistical significance was the same when we used the unpaired t-test to log-transformed data. * Differences were considered significant at *p <* 0.05 (*n* = 6).

We observed a shift in the microbiota composition at phylum levels (Figure 3a). At the phylum level, the relative abundance of *Firmicutes*/*Bacaeroides* was significantly larger in the OT and MT groups compared to the Con and AB groups (*p <* 0.05, Figure 3b). The relative abundance of *Proteobacteria* was significantly less in the MT group compared to the Con group (*p <* 0.05, Figure 3c), which was confirmed by Mann–Whitney tests for both phyla. There were no significant differences in both community richness (Chao1) and diversity index (Shannon) among the experimental groups (*p >* 0.05, Figure 3d,e).

### 3.6. Metabolites of the Intestinal Contents

**Figure 4:**
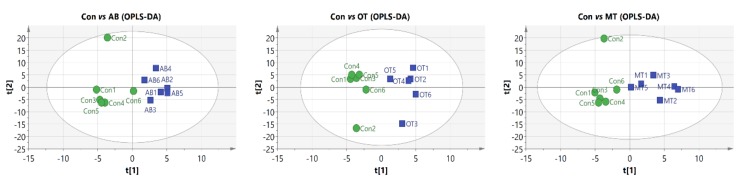
Gas chromatography time-of-flight mass spectrometry (GC-TOF-MS) and metabolomics analysis. Orthogonal projections to latent structures-discriminant analysis (OPLS-DA) score map derived from the GC/MS metabolite profiles of intestinal contents (*n* = 6).

The metabolomics analysis showed that a total of 914 spectral peaks were identified as reliable metabolites through comparison with the LECO/Fiehn Metabolomics Library. A clear separation and discrimination were found among the AB, OT and MT groups by OPLS-DA analysis (Figure 4).

The levels of 6, 8 and 8 metabolites identified in the intestinal contents from AB, OT and MT group respectively were significantly changed compared to the Con group (Table 7, Table 8 and Table 9). Six metabolites that increased in AB treatment compared with the Con were benzamide, dihydrocholesterol, N-acetylornithine, hypoxanthine, galactitol, trans-4-hydroxyproline (Table 7). The treatment of MT resulted in a significant increase in metabolites, which were catechol, isoleucine, acetoacetate, trisaccharide, lactic acid, serine and phenylalanine (Table 9).

### 3.7. Correlation Analysis of Different Flora and Metabolites

Based on the intestinal flora participating in the host’s nutritional metabolism function, the Pearson correlation coefficients between the 11 different phylum levels of flora and 22 differential metabolites (VIP > 1.5, *p <* 0.05) were further calculated. A correlation network was constructed for related flora and differential metabolites with correlation coefficients greater than 0.41 or less than −0.41 (Table 10).

## 4. Discussion

Supplementation of tributyrinin the diet was able to provide butyric acid for the intestine tissuestodirectly use as nutrients. The fragrances of oregano and methyl salicylate can increase the appetite of the animals, activate their gastrointestinal receptors and stimulate the activity of digestive enzymes, thereby enhance the absorption of nutrients and promote the growth of the body [29]. A previous study found that supplementation of 0.5% TB in their diet significantly improved daily weight gain and reduced the ratio of feed intake/weight gain in weaned piglets [30]. However, in a different study it was found that TB at a dosage of 1% in their diet negatively affected the performance of weanling pigs [31]. Additionally, previous research showed that treatment of oregano oil had no improvement effect on growth performance of nursery pigs [32]. In this study, the dietary inclusion of OT and MT as well as AB had a numerical improvement on growth performance of the piglets, although the differences between these groups and the Con were not significant. Considering the current study was mainly designed to test the effect on gut health and microbiota and not for growth performance, more trials are needed to test their effect on growth performance in the future.

Intestinal morphology is an important factor reflecting intestinal health and function in pigs [33]. Weaning could cause gastrointestinal tract dysfunction in piglets, which is often due to the contraction of small intestine villi, the increase of crypt depth and the decrease of enzyme activity. These changes in intestinal morphology can cause diarrhea, reduce feed intake and delay the animal’s growth [34]. In this study, dietary inclusion of OT or MT significantly increased the villus height in the duodenum of piglets. A previous study found that dietary treatment with tributyrin in piglets with intrauterine growth retardation (IUGR) could significantly increase villus length and decrease crypt depth in the duodenum and jejunum [4]. In different piglet studies it was found that dietary sodium butyrate increased the height of the duodenum villi and the depth of the crypt [35], whereas inulin-coated sodium butyrate resulted in a 25% larger height of villi of the ileum compared to the control [36]. Conversely, a study found oral administration of sodium butyrate decreased the jejunum mucosa thickness and villous height [37]. Also, several studies have found that dietary sodium butyrate treatment has no effect on intestinal mucosal morphology or jejunal villus height in weaned piglets [38,39]. In general, our current study suggests that dietary inclusion of the combination of tributyrin and oregano or methyl salicylate effectively promotes the intestinal morphological structure with more healthy characteristics.

The beneficial effect of oregano may be attributed to carvacrol, which contributes to maintaining a good intestinal ecosystem in weaned piglets [40]. Additionally, its components include carparinol and thyme, which are beneficial to intestinal mucosa and accelerate the regeneration rate of intestinal villi surface epithelial cells [41]. Methyl salicylate is also an essential oil which has many healthy attributes including antioxidation and anti-inflammation effects. So far, little information is available on the possible effect of methyl salicylate on the intestinal structure of piglets. This study, that showed improving effects on piglet intestine health by dietary inclusion of a combination of tributyrin with oregano or methyl salicylate, confirmed our hypothesis on their beneficial functions.

The dynamic balance of intestinal microbes is an important prerequisite for the health of piglets [42]. When the pH is greater than 4, harmful bacteria in the digestive tract increase while the beneficial bacteria decrease; when the pH value is less than 4, the beneficial bacteria increase while the harmful bacteria decrease [43]. The addition of tributyrin and essential oils to the feed of piglets reduced the pH of the gut, which might lead to well-balanced intestine microflora. In the bacterial cell, butyric acid is broken down into hydrogen and butyrate ions. The increased concentration of hydrogen ions negatively affects the growth of harmful bacteria such as *Escherichia coli* and *Salmonella*, but has little effect on beneficial bacteria such as *Lactobacillus,* which is acid-resistant [44]. A previous study found that inclusion of 0.3% butyric acid in the diet of early weaned piglets significantly increased the diversity of intestinal microorganisms [45]. Another study found that supplementation of 0.4% or 0.6% sodium butyrate also reduced the pH in the stomach and duodenum, and resulted in a reduced number of harmful bacteria in the stomach and cecum [46].

The essential oils contain bactericidal active components which have strong lipid solubility and surface activity. They can rapidly penetrate the cell membrane of pathogenic bacteria. On the other side, the essential oils might also inhibit the bacteria through a block respiratory oxidation process in mitochondria [47]. A previous study found that a mixture of thyme and parsley cheese reduced the number of *Escherichia coli* but increased that of *Lactobacillus* in the feces of weaned piglets [48], which was similar to the results found in the current study in the OT and MT groups.

Studies have found that intestinal microbes are mainly anaerobic bacteria [49]. Through the level analysis on the flora structure in pigs it was found that the dominant bacteria in the intestine were mainly *Bacteroides* and *Firmicutes* [50,51]. Intestinal *Firmicutes* and *Bacteroides* degrade polysaccharides and promote energy absorption, and therefore the ratio of *Firmicutes* to *Bacteroidetes* could be of significant relevance to gut microbiota status [52]. *Proteobacteria* include many bacteria which might be harmful to intestinal health, such as *Escherichia coli*, *Salmonella* and *Helicobacter pylori*. The abundance of *Proteobacteria* is related to the intestinal microbial community and metabolism. Normally, a *Proteobacteria*-dominated community is commonly observed in the intestinal tract of organisms with malnutrition and metabolic disorders. Moreover, a *Proteobacteria*-dominated community is likely to cause immune disorders in the body which could further lead to intestinal inflammation [53]. In the current study it was found that dietary tributyrate combined with oregano oil or methyl salicylate significantly increased the relative abundance of *Firmicutes* and decreased the relative abundance of *Proteobacteria*, *Actinobacillus* and *Escherichia*, which indicated that both combined treatments exerted a beneficial influence on the development of healthy intestinal flora.

Metabolomic studies on intestinal content may provide more information regarding the effect of dietary treatment on intestinal health and microbiota. The current study found that the dietary treatments resulted in significant changes in some important metabolites i.e., the OT treatment reduced carnitine and the MT treatment increased 3-aminoisobutyric acid. Carnitine is an important metabolite involved in fatty-acids metabolism [54] whereas 3-aminoisobutyric acid provides energy for cellular metabolism [55]. Lactic acid could form acetylated acid and enter mitochondria for oxidative phosphorylation. Moreover, it could promote the differentiation and development of bone cells [56,57]. The changes in the intestinal metabolites suggest the dietary treatments of OT and MT might affect nutrient digestion and metabolism as well as the profile of intestinal microbiota.

Microbial community diversity and metabolomics analysis may be new indicators of intestine stability and functions which are closely related to animal health; together, the analysis offers a promising approach to evaluate the functional status in the interaction between microbiota and the host intestine [58]. In the current study, both the microbiota and metabolomics-based analysis were used to evaluate the effect of dietary tributyrin combined with oregano or methyl salicylate on the intestinal microbial society in weaned piglets. Through Pearson analysis on the flora and the major affected metabolites, the study found there were high positive correlations between amounts of *Firmicutes* and dihydrocholesterol, as well as between amounts of *Proteobacteria* and mannonic acid. Such results on the correlation analysis between intestinal flora and metabolites may provide more evidence that the intestinal flora plays important roles in animal health.

## 5. Conclusions

The inclusion of a combination of tributyrin with oregano or methyl salicylate to the diet of weaned piglets increased the ratio of villus height to crypt depth in the intestine of piglets and resulted in major changes in the gut profiles of microbiota and metabolites, which was beneficial to the animal health. The results indicated that the combination of tributyrate with oregano or methyl salicylate could be a potential candidate as an alternative feed additive to antibiotics.

## Figures and Tables

**Table 1:** Composition and nutrient contents of the basal diets.

Items	Basal Diets
Ingredients %	
Corn	61.0
Soybean meal	10.4
Fermented soybean meal	8.0
Soybean oil	2.5
Extruded soybean	7.0
Imported fish meal	3.4
Whey powder	2.7
Premix ^1^	5.0
Nutrient levels ^2^	
Digestible energy (MJ/kg)	14.68
Crude protein	18.84
Calcium	0.65
Phosphorus	0.56
Lysine	1.30
Methionine	0.78
Threonine	0.85
Tryptophan	0.22

^1^ The pre-mixed mixture provided for piglets (per kg of diet): Cu, 180 mg; Fe, 120 mg; Zn, 80 mg; Mn, 40 mg; I, 1 mg; Se, 0.25 mg; Co, 0.4 mg; vitamin A, 22,500 IU; vitamin D_3,_ 4950 IU; vitamin E, 89 IU; vitamin K_3_, 2.7 mg; vitamin B_1_, 5.2 mg; vitamin B_2_, 15 mg; vitamin B_6_, 8.1 mg; vitamin B_12_, 4.5 mg; pantothenic acid, 42 mg; niacin, 52 mg; folic acid, 3.3 mg; biotin, 0.3 mg; choline chloride, 1500 mg. ^2^ The nutrient contents of crude protein, calcium, and phosphorus were the examined values, and the contents of other nutrients were calculated.

**Table 2:** Effect of dietary supplementation with tributyrin in combination with different essential oils on growth performance of weaned pigs.

Items	Con ^1^	AB^1^	OT^1^	MT ^1^	SEM ^2^	*p*-Value
Initial body weight, kg	8.69	8.95	8.98	8.54	0.483	-
Final body weight, kg	15.85	17.44	16.88	16.78	0.823	0.152
Daily intake, kg	0.520	0.581	0.560	0.563	0.015	0.223
Average daily gain, kg	0.256	0.303	0.282	0.294	0.014	0.153
Feed to gain ratio, kg/kg	2.043	1.922	1.985	1.927	0.080	0.205

^1^ Con, the basal diet without antibiotics, tributyrin, oregano or methyl salicylate; AB diet, the basal diets supplemented with an antibiotic compound, which provides the diet with 120 mg oxytetracycline-calcium and 16 mg enduracidin per kilogram; OT and MT diets, the basal diet supplemented with tributyrin plus oregano (1.4 g and 0.6 g per kilogram diet) and tributyrin plus methyl salicylate (1.4 g and 0.6 g per kilogram diet), respectively. Data are means of three replicate pens; ^2^ SEM, standard error of the mean.

**Table 3:** Effect of dietary supplementation with tributyrin in combination with different essential oils on serum characteristics of weaned pigs.

Items ^2^	Con ^1^	AB ^1^	OT ^1^	MT ^1^	SEM ^3^	*p*-Value
Total protein, g/L	44.3	42.8	42.1	41.7	1.69	0.80
Albumin, g/L	24.2	23.3	23.3	23.5	1.25	0.95
Glucose, mmol/L	4.0	5.0	5.0	4.7	0.32	0.21
BUN, mmol/L	1.83	1.97	1.97	2.34	0.28	0.64
AST/GOT, U/L	95.0	66.7	64.3	86.0	10.97	0.19
ALT/GPT, U/L	90.4	85.7	83.6	97.6	9.47	0.74
ALP, mmol/L	14.1	15.7	14.0	17.0	1.08	0.25
SOD, U/mL	94.2	91.8	90.5	93.7	2.08	0.58
MDA, nmol/mL	4.0	3.3	4.0	3.6	0.35	0.50
GSH-PX, U/mL	116.2	112.4	111.7	113.4	3.25	0.79
T-AOC, mmol/L	0.28	0.23	0.23	0.26	0.02	0.36

^1^ Con, the basal diet without antibiotics, tributyrin, oregano or methyl salicylate; AB diet, the basal diets supplemented with an antibiotic compound, which provides the diet with 120 mg oxytetracycline-calcium and 16 mg enduracidin per kilogram; OT and MT diets, the basal diet supplemented with tributyrin plus oregano (1.4 g and 0.6 g per kilogram diet) and tributyrin plus methyl salicylate (1.4 g and 0.6 g per kilogram diet), respectively; ^2^ BUN, blood urea nitrogen; AST/GOT, glutamic oxalo-acetic transaminase; ALT/GPT, glutamic pyruvic transaminase; ALP, alkaline phosphatase; SOD, super oxide dismutase; MDA, model driven architecture; GSH-PX, glutathione peroxide; T-AOC, total anti-oxidation capacity; ^3^ SEM, standard error of the mean (*n* = 6).

**Table 4:** Effect of dietary supplementation with tributyrinin combination with different essential oils on pH value of the gastrointestine of weaned pigs.

Items	Con ^1^	AB ^1^	OT ^1^	MT ^1^	SEM ^2^	*p*-Value
Stomach	3.22	3.29	2.82	2.66	0.349	0.614
Duodenum	5.84	5.02	4.82	4.53	0.355	0.103
Jejunum	6.53	6.22	6.07	6.13	0.137	0.143
Ileum	6.56 ^a^	6.19 ^a,b^	6.38 ^a^	5.63 ^b^	0.160	0.006
Colon	5.81	5.68	5.83	5.82	0.134	0.861
Cecum	5.73	5.60	5.81	5.70	0.090	0.497

^1^ Con, the basal diet without antibiotics, tributyrin, oregano or methyl salicylate; AB diet, the basal diets supplemented with an antibiotic compound, which provides the diet with 120 mg oxytetracycline-calcium and 16 mg enduracidin per kilogram; OT and MT diets, the basal diet supplemented with tributyrin plus oregano (1.4 g and 0.6 g per kilogram diet) and tributyrin plus methyl salicylate (1.4 g and 0.6 g per kilogram diet), respectively; ^2^ SEM, standard error of the mean; ^a,b^ Means without a common superscript letter differ (*p <* 0.05)(*n* = 6).

**Table 5:** Effect of dietary supplementation with tributyrin in combination with different essential oils on short-chain fatty acid in the colon of weaned pigs.

Items	Con ^1^	AB ^1^	OT ^1^	MT ^1^	SEM ^2^	*p*-Value
Acetic acid, μg/mL	32.3	36.6	30.0	38.5	2.31	0.067
Propionic acid, μg/mL	12.7	14.4	10.5	15.6	1.46	0.116
Isobutynic acid, μg/mL	0.49	0.40	0.53	1.16	0.35	0.413
Butynic acid, μg/mL	9.85	9.22	8.79	9.58	1.20	0.932
Isovaleric acid, μg/mL	0.70	0.46	0.66	1.20	0.34	0.472
Valeric acid, μg/mL	0.95	0.79	0.76	0.91	0.10	0.440

^1^ Con, the basal diet without antibiotics, tributyrin, oregano or methyl salicylate; AB diet, the basal diets supplemented with an antibiotic compound, which provides the diet with 120 mg oxytetracycline-calcium and 16 mg enduracidin per kilogram; OT and MT diets, the basal diet supplemented with tributyrin plus oregano (1.4 g and 0.6 g per kilogram diet) and tributyrin plus methyl salicylate (1.4 g and 0.6 g per kilogram diet), respectively; ^2^ SEM, standard error of the mean (*n* = 6).

**Table 6:** Effect of dietary supplementation with tributyrin in combination with different essential oils on small intestinal morphology of weaned pigs.

Items	Con ^1^	AB ^1^	OT ^1^	MT ^1^	SEM ^2^	*p*-Value
*Duodenum*						
Villus height, μm	637 ^a^	741 ^a,b^	776 ^b^	822 ^b^	31.3	0.008
Crypt depth, μm	186	189	196	182	10.4	0.839
VH/CD	3.44 ^a^	3.97 ^a,b^	3.96 ^a,b^	4.59 ^b^	0.20	0.015
*Ileum*						
Villus height, μm	503	519	542	534	29.8	0.838
Crypt depth, μm	127 ^a^	152 ^b^	122 ^a^	124 ^a^	2.9	0.001
VH/CD^3^	3.97 ^a,b^	3.40 ^a^	4.43 ^b^	4.31 ^b^	0.20	0.011

^1^ Con, the basal diet without antibiotics, tributyrin, oregano or methyl salicylate; AB diet, the basal diets supplemented with an antibiotic compound, which provides the diet with 120 mg oxytetracycline-calcium and 16 mg enduracidin per kilogram; OT and MT diets, the basal diet supplemented with tributyrin plus oregano (1.4 g and 0.6 g per kilogram diet) and tributyrin plus methyl salicylate (1.4 g and 0.6 g per kilogram diet), respectively; ^2^ SEM, standard error of the mean; ^3^ VH, villus height; CD, crypt depth; ^a,b^ Means without a common superscript letter differ (*p <* 0.05) (*n* = 6).

**Table 7:** List of significantly changed metabolites between the Con group and the AB group.

Metabolite	R.T. (min) ^1^	Mass	Similarity	VIP ^2^	*p*-Value	FC ^3^
Benzamide	15.46	333	888.74	2.40	0.020	0.38
Dihydrocholesterol	6.33	130	922.19	1.54	0.031	0.12
N-acetylornithine	8.45	147	928.85	1.83	0.046	0.53
Hypoxanthine	5.99	156	921.60	1.60	0.050	0.37
Galactinol	5.90	147	827.07	2.26	0.053	0.87
Trans-4-hydroxyproline	13.19	217	904.07	1.58	0.057	0.28

Con, the basal diet without antibiotics, tributyrin, oregano or methyl salicylate; AB diet, the basal diets supplemented with an antibiotic compound, which provides the diet with 120 mg oxytetracycline-calcium and 16 mg enduracidin per kilogram; ^1^ R.T. represents the retention time; ^2^ VIP = variable importance projection metabolite as listed in table; ^3^ FC represents the fold change of the peak intensity for the control group against the AB group (*n* = 6).

**Table 8:** List of significantly changed metabolites between the Con group and the OT group.

Metabolite	R.T.(min) ^1^	Mass	Similarity	VIP ^2^	*p*-Value	FC ^3^
Glutamic acid	16.1	147	892.88	1.50	0.015	85.35
5-hydroxy-3-indoleacetic acid	18.63	204	776.72	1.51	0.019	35.19
Xylose	18.27	204	677.98	1.59	0.030	3.03
Mannonic acid	8.77	245	950.96	1.79	0.043	1.79
Pyruvic acid	8.08	142	955.29	1.52	0.049	3.69
3-aminoisobutyric acid	23.26	204	837.25	1.68	0.051	10.31
Ethanolamine	10.5	147	917.71	1.77	0.054	2.76
Carnitine	9.75	258	872.25	1.50	0.054	4.15

Con, the basal diet without antibiotics, tributyrin, oregano or methyl salicylate; OT, the basal diet supplemented with tributyrin plus oregano (1.4 g and 0.6 g per kilogram diet); ^1^ R.T. represents retention time; ^2^ VIP = variable importance projection metabolite as listed in table; ^3^ FC represents the fold change of the peak intensity for the control group against the OT group (*n* = 6).

**Table 9:** List of significantly changed metabolites between the Con group and the MT group.

Metabolite	R.T.(min) ^1^	Mass	Similarity	VIP ^2^	*p*-Value	FC ^3^
Catechol	21.19	371	878.69	1.90	0.010	0.49
Isoleucine	18.16	129	874.99	1.79	0.017	0.46
Acetoacetate	18.21	117	938.18	2.00	0.017	0.44
Trisaccharide	16.64	117	978.94	1.76	0.023	0.51
Lactic acid	18.43	117	915.83	1.57	0.026	0.52
Serine	17.55	117	882.94	1.82	0.030	0.45
Phenylalanine	13.16	117	896.50	1.60	0.037	0.05
3,5-dihydroxyphenylglycine	4.98	128	900.81	2.03	0.060	2.46

Con, the basal diet without antibiotics, tributyrin, oregano or methyl salicylate; MT diets, the basal diet supplemented with tributyrin plus methyl salicylate (1.4 g and 0.6 g per kilogram diet); ^1^ R.T. represents retention time; ^2^ VIP = variable importance projection metabolite was listed in table; ^3^ FC represents the fold change of the peak intensity for the control group against the MT group (*n* = 6).

**Table 10:** Interaction of characteristic floras and metabolites based on Pearson correlation coefficient.

Items	Metabolite	Correlation	*p*-Value
Firmicutes	Dihydrocholesterol	0.499	0.0165
	Hypoxanthine	0.432	0.0408
	Mannonic acid	−0.587	0.0038
	Catechol	0.490	0.0188
	Acetoacetate	0.423	0.0455
Proteobacteria	Dihydrocholesterol	−0.422	0.0461
	Mannonic acid	0.592	0.0035
Bacteroidetes	3,5-dihydroxyphenylglycine	−0.445	0.0332
Tenericutes	Dihydrocholesterol	−0.582	0.0042
	5-hydroxy-3-indoleacetic acid	0.453	0.0314
	3-aminoisobutyric acid	0.432	0.0408
	Phenylalanine	−0.562	0.0111
Fusobacteria	3,5-dihydroxyphenylglycine	−0.571	0.0045
Cyanobacteria	Dihydrocholesterol	0.635	0.0011
	Hypoxanthine	0.554	0.0061
	Mannonic acid	−0.440	0.0355
	Catechol	0.518	0.0113
	Acetoacetate	0.422	0.0451
Actinobacteria	Hydroxyproline	−0.463	0.0262
	Phenylalanine	0.485	0.0301
Euryarchaeota	Benzamide	−0.432	0.0396
	Galactinol	−0.574	0.0042
	Mannonic acid	−0.418	0.0472
Saccharibacteria	Benzamide	−0.511	0.0127
